# Comparative anticancer effects of *Annona muricata* Linn (Annonaceae) leaves and fruits on DMBA-induced breast cancer in female rats

**DOI:** 10.1186/s12906-023-04073-x

**Published:** 2023-07-15

**Authors:** Kevine Kamga Silihe, William Defo Mbou, Judith Christiane Ngo Pambe, Larissa Vanelle Kenmogne, Laure Fotso Maptouom, Marius Trésor Kemegne Sipping, Stéphane Zingue, Dieudonné Njamen

**Affiliations:** 1 Department of Biochemistry, Faculty of Science, University of Yaoundé 1, P.O. Box 812, Yaoundé, Cameroon; 2 Department of Animal Biology and Physiology, Faculty of Science, University of Yaoundé 1, P.O. Box 812, Yaoundé, Cameroon; 3 Department of Morphological Sciences and Pathological Anatomy, Faculty of Medicine and Biomedical Sciences, University of Garoua, P.O. Box 317, Garoua, Cameroon; 4 Department of Pharmacotoxicology and Pharmacokinetics, Faculty of Medicine and Biomedical Sciences, University of Yaounde 1, P.O. Box 1364, Yaounde, Cameroon

**Keywords:** *Annona muricata* Linn, Breast cancer, Functional food, DMBA, CA15-3, Cytokines

## Abstract

**Background:**

Numerous studies have reported the anti-cancer effects of different parts of *Annona muricata* Linn, however ; most of them focused on the in vitro evaluation of isolates. In vivo evidence on which part is best suited for breast cancer chemoprevention remains to be demonstrated. This is a comparative study of the effects of *A. muricata* fruit and leaves extracts on DMBA induced-breast cancer in rats.

**Methods:**

Rats exposed to DMBA (50 mg/kg, *s.c.*), were treated with *A. muricata* fruit aqueous extract at 200 mg/kg BW (3 days/week or daily) and *A. muricata* Linn leaves ethanolic extract at 200 mg/kg daily. Positive control group received tamoxifen at 3.3 mg/kg, while the normal and diseased controls received vehicle. After 20 weeks of treatment, the tumor incidence, tumor burden, tumor volume, histopathology, protein and CA 15 − 3 levels as well as antioxidant status, pro-inflammatory cytokines were assessed.

**Results:**

Thus, 100% of diseased rats presented cribriform ductal carcinoma of SBR grade III. *A. muricata* extracts (leaves and fruit) and tamoxifen significantly reduced death and tumor incidences, volume and weight of the tumors, total protein and CA15-3 levels compared to the DMBA group. They exhibited antioxidant activity, through an increase in the GSH level and SOD and catalase activities with reduced levels of MDA compared to DMBA group. TNF-α, IL-6 and INF-γ levels reduced with regards to *A. muricata* treatment.

**Conclusion:**

These results confirm the anti-breast cancer effect of *A. muricata*, however, the aqueous fruit extract was more potent than the ethanolic leaves extract.

## Background

Cancer is a large family of diseases that involves abnormal cell growth with the potential to invade or spread to other parts of the body [1]. It is a major health problem and the second leading cause of mortality worldwide after heart diseases; 19.3 million new cases and 10 million deaths were recorded in 2020 [2]. Cancer Research UK predicts that there will be 27.5 million new cases of cancer globally by 2040 and nearly 70% of cases will occur in developing countries if the current trend persists [3]. In females, the four most common types of cancer are breast cancer, colorectal cancer, lung cancer and cervical cancer [4]. Amongst these four, breast cancer accounts for 30% of all new cancer cases and was the leading cause of cancer related deaths (685,000 deaths) in 2020 [5]. In Africa, it is the leading cause of death among women which accounts for 28% of all cancers and 20% of all cancer deaths in women [2]. Women in Cameroon are not excluded from this major public health challenge which accounts for more than 4170 (20.1%) new cases and 2108 (16%) deaths per year [4].

The etiology of breast cancer remains unclear, but factors such as smoking, obesity, inadequate diet, lack of physical activity, pollution (exposure to polycyclic aromatic hydrocarbons-PAHs such as DMBA), genetic mutations (BRCA1 and BRCA2 tumor suppressor genes) and estrogen impregnation (early menarche and late menopause) increase the risk of breast cancer [6]. Many treatments directed against breast cancer are now available, some examples include; chemotherapy that can be used alone or in combination with surgery, radiotherapy, hormone therapy, and targeted therapy [7]. However, these therapies do not absolutely reverse metastatic breast cancer and are laden with many other drawbacks including: chemotherapy drug toxicity on healthy cells (paclitaxel); endometrial cancer risk (tamoxifen) and cardiotoxicity (doxorubicin) [8]. In addition, in Africa, these treatments are limited by high-cost, multidrug resistance and tumor relapse [9]. Given the adverse effects of conventional therapy, interest in alternative or complementary medicine, particularly phytotherapy has attracted considerable attention [10]. Natural compounds which are effective, less costly and less toxic against breast cancer are readily available in plants among which *Annona muricata* Linn [11].

*Annona muricata* Linn (common name “soursop”) is a plant widely distributed in tropical regions and found in Cameroon. It has many virtues and is known to possess biological properties such as sedative, bactericidal, antiparasitic, anti-inflammatory, astringent, hemostatic, antispasmodic, anti-ulcer, anti-diarrhea, antitussive, hypotensive, antipyretic, galactogogue and anticancer [12]. All over the world, this plant is used conventionally to treat cancer [13] and numerous studies have demonstrated its cytotoxic, cytostatic, anti-cancer effects in vitro and in vivo on various tissues as well as its underlying mechanisms [14, 15]. Minari and Okeke [16], have shown that *A. muricata* Linn leaf extract has chemoprotective effect in DMBA-induced breast neoplasia in albino mice. *A. muricata* L. fruit extract strongly abrogated the MDA-MB-468 cells growth (IC_50_ = 4.8 µg/mL) *via* cell cycle arrest, apoptosis and EGFR downregulation. Furthermore, it downregulated the EGFR, p-EGFR, and p-ERK proteins in tumor’s xenograft mouse model (MDA-MB-468) after 5-weeks of dietary treatment at 200 mg/kg [17]. Compounds isolates from *A. muricata* leaves and fruits extracts inhibited tumorigenicity and metastasis in vitro and in vivo in rats by altering cell metabolism [18]. Phytochemical investigation of *A. muricata* Linn has been extensively studied and it was found to be rich in acetogenins and alkaloids which are responsible for its anticancer potentials. Acetogenis from *A. muricata* exhibited potent anticancer effects (IC_50_ = 14.69 µM) on multidrug resistant breast tumors *via* an alteration of mitogen-activated protein kinase (MAPK) signaling and induction of apoptosis in MCF-7/ADR cells *via* the mitochondrial pathway [19]. Nevertheless; most of these studies have been concerned with the in vitro anti-cancer evaluation of its isolates on various cancer cell lines. Very little reports found in the literature dealt with its in vivo anticancer effects on breast. Taking into account the fact that drug metabolism could affect efficacy of a substance, the in vivo assessment on its effects in a DMBA-induced breast cancer in Wistar rats is a current need. The present study therefore aimed to shed more light on which of the two most effective parts (leaves or fruit) according to in vitro studies might be better suited to prevent DMBA induced-breast cancer in rats.

## Methods

### Chemicals and reagents

The breast carcinogen 7,12 dimethylbenz(a)anthracene (DMBA, purity ≥ 98%), was obtained from Sigma-Aldrich (Stanford, Germany), while the tamoxifen citrate (Norvatis Access®) was obtained from Salutas Pharma GmbH (Barbelen, Germany). The anesthetics, diazepam (Valium® 10 mg/2 ml) and ketamine (Ketamine hypochloride® 50 mg/ml) were obtained from Roche (Fontenay-sous-bois, France) and Rotex Medica (Tritau, Germany), respectively. The cytokine kit MILIPLEX® for lumiex® Xmap® Technology was obtained from Milipore (R&D systems, inc, Minneapolis, USA). Cancer antigen 15 − 3 (CA 15 − 3) enzyme-linked immunosorbent assay (ELISA) kit was obtained from Monobind Inc® (California, USA). The alanine transaminase (ALT) and creatinine reagent kits were from Fortress Diagnostics Limited® (Muckamore, United Kingdom).

### Plant

#### Collection and authentication of the plant

The leaves and fruits of *A. muricata* Linn were collected at Ngoa-Ekélé in Yaoundé (Center region, Cameroon). The sample used in this study was authenticated at the National Herbarium of Cameroon (HNC) in Yaounde by Dr. Damien Essono (Botanist in Faculty of Science, University of Yaounde I). A voucher specimen (N° 32,879 NHC) was registered and deposited for ready reference at the HNC. The plant name has been checked with http://www.theplantlist.org; consulted the 27 April 2022.

#### Preparation of A. muricata Linn pulp fruit extract

*A. muricata* Linn fruits were harvested and kept for 8 days to mature. They were cleaned, stripped of their seeds and then crushed in a blender. The resulting paste (2.8 g) was sieved and filtered using Whattman paper N° 4. Afterward, the filtrate (3 L) obtained was dried in an oven for 48 h at 45 °C. After drying, 87 g of *A. muricata* Linn aqueous extract (2.9% yield) was obtained and referred to AMF.

#### Preparation of A. muricata Linn leaves ethanolic extract

After being cleaned and dried in a shade, a propeller mill was used to grind the leaves into powder (1500 g). The powder was macerated in 6 L of 95% ethanol for 48 h at room temperature. The process was repeated twice and 32 g (2.13%) of crude ethanolic leaves extract (AML) was obtained after filtration through Whatman paper N° 4 and evaporation on a rotary evaporator under reduced pressure at 175 mbar at 40 °C.

### Characterization of A. muricata extract

#### Proximal analysis

The water content in *A. muricata* Linn juices was determined following AFNOR [20] protocol’. Briefly, 0.5 g of fresh extracts sample were dried at 105 °C in an oven for 24 h. The result was expressed as a percentage by subtracting the dry matter content from 100.

#### Determination of phenols content

The total phenol content in *A. muricata* Linn extracts was assessed by Folin-Ciocalteu method [21] with gallic acid as standard. Thus, 75 µL of freshly prepared Folin-Ciocalteu reagent was mixed with 750 µL of sample concentrate (1 mg/mL). Then 3 min later, 750 µL of Na_2_CO_3_ (20%) was added to the mixture and incubated for 30 min in the dark. The absorbance was measured at 760 nm using a UV-VIS 1605 Shimadzu spectrophotometer.

#### Estimation of flavonoids content

The flavonoid content in *A. muricata* extracts was estimated following the Chang et al. [22]. protocol with slight modifications and quercetin served as standard. For this, a reacting solution was prepared by mixing 1 mL of sample concentrate (1 mg/mL) plus 100 µL of 1 M sodium acetate and 100 µL of 10% aluminum chloride. After 40 min of incubation at room temperature the absorbance was read at 415 nm with a UV-VIS 1605 Shimadzu spectrophotometer.

#### DPPH (1,1-Diphenyl-2-picryl hydrazyl) assay

*A. muricata* Linn extracts free radicals scavenging of DPPH was determined by their ability to donate hydrogen [23]. Indeed, 400 𝜇mol/L of DPPH in methanol was prepared just before use and 500 𝜇L of the solution was added to *A. muricata* Linn extracts (100 − 300 𝜇g/mL) or ferulic acid (positive control). The solution was incubated in the dark at 37 °C for 30 min and absorbance was measured at 517 nm using a Shimadzu UV-1700 spectrophotometer. The DPPH radical scavenging effect or “inhibition percentage” was calculated: Percentage of inhibition (%) = {[(𝐴0− 𝐴1)/𝐴0] × 100} where 𝐴0 is the absorbance of the control reaction and *A*1 is the absorbance of the sample.

#### FRAP

The reducing potential was determined using FRAP method [24]. The FRAP solution (300 mmol/L sodium acetate buffer (pH 3.6), 10 mmol/L TPTZ solution in 40 mmol/L HCl, and 20 mmol/L FeCl_3_·6H_2_O solution at a ratio of 10:1:1 (v/v)) and 0.15 mL of diluted sample (0.5 mg/ml) were mixed and incubated at 37 °C in the dark for 30 min. The optical density of the colored product (ferrous tripyridyltriazine complex) was read at 593 nm. Data are presented as mg of quercetin equivalent (AAE)/g extract.

### DMBA-induced breast cancer in rat

#### Experimental animals

Prepubertal female Wistar rats aged 35 to 40 days and weighing ~ 55 g at the beginning of the experiment were obtained from the animal house of the Animal Physiology Laboratory, Faculty of Science, University of Yaoundé I. Rats were allowed in plastic cages at room temperature (~ 25 °C) with sufficient ventilation under a natural light cycle. They had access to tap water *ad libitum* and standard soy-free rat chow. The constituents of the diet were: corn (42%), bone meal (3%), wheat flour (22%), fish meal (19%), crushed palm kernel meal (4%), sodium chloride (0.75%), peanuts (9%) and multivitamin complex (Olivitazol® 0.5%).

Animal handling and treatments were approved by the Joint Institutional Review Board on Animal & Human Bioethics of the Faculty of Science (University of Yaoundé 1) reference # BTC-JIRB2021-010, which is in line with the European Union on the care of animals (EEC Council 86/609).

#### Dose determination

Mammary tumors were induced in prepubertal rats (~ 55 days) using the carcinogen DMBA (50 mg/kg BW) dissolved in 1 ml of olive oil, (rats in the normal group received olive oil only) [25]. Tamoxifen (Tamox, 3.3 mg/kg BW), a selective estrogen receptor modulator (SERM) was used as standard [26]. *A. muricata* Linn leaves extract (AML) was tested at the optimal dose of 200 mg/kg BW [27]. *A. muricata* pulp fruit extract (AMF) was administered at 200 mg/kg BW at 2 different frequencies (3 days/week and daily).

#### Treatment of rats

To achieve our goal, forty-height (48) healthy female Wistar rats aged 35–40 days were allowed for 10 days of acclimatization and then randomly distributed at the age of 45–50 days in labeled plastic cages. The experiment consisted of 6 groups of 8 animals each (n = 8): groups 1 (NOR) and 2 (DMBA) were administered distilled water (vehicle) and served as normal and negative control groups, respectively; group 3 (Tamox) received tamoxifen (3.3 mg/kg) and served as the positive control group; group 4 received *A. muricata* Linn leaves ethanolic extract (AML) at the dose of 200 mg/kg, while groups 5 and 6 received *A. muricata* Linn pulp fruit extract (AMF) at the dose of 200 mg/kg BW at 2 different frequencies (3 times/week or every day). Treatments lasted 20 weeks and were done through gavage.

#### Organ collection

In the course of the study, weighing of the rats was done weekly and they were palpated twice a week to detect mammary tumors. Animals that became moribund or died were directly sacrificed and/or autopsied. After 20 weeks, surviving animals were sacrificed by decapitation under anesthesia after 12 h of fasting. Blood samples were collected in anticoagulant tubes (ethylenediaminetetraacetic-EDTA) and in dry tubes (centrifuged at 600 × g for 15 min at 4 °C) for hematological and biochemical analysis, respectively. Further, breast tumors were removed, counted, weighed and measured with a 1 mm precision caliper (IGAGING®). Breast tumors, mammary glands, liver, spleen, lungs and kidneys were also collected, weighed and immediately fixed in 4% formol for histological analysis.

Tumor volume was deduced from the formula (length × weight × height × π / 6) of Faustino Rocha et al. [28]. The relative organs weight was calculated as follows: relative organs weight (mg/kg) = [organ weight (mg)/rat body weight (kg)] × 10^6^. The tumor incidence was calculated as follows: tumor Incidence (%) = (number of rats with tumors/ total number of rats) × 100. The tumor burden was estimated as follows: tumor burden (g) = sum of the total relative tumor mass in a group. The % inhibition of tumor burden was obtained as follows: % inhibition of tumor burden = (tumor burden in DMBA - tumor burden in test group)/tumor burden in DMBA) × 100.

### Histological analysis

Collected organs were subjected to fundamental histological techniques consisting of trimming, dehydration and inclusion. The 5-µm sections of paraffin-embedded tissues were stained using the classical hematoxylin–eosin stain and mounting under resin. The changes in tumors and organs’ histoarchitecture were assessed on photomicrographs obtained by using a light Axioskop 40 microscope equipped with a digital Celestro-44,421 camera connected to a computer where the images were transferred and analyzed with Image J software. The breast tumors were analyzed in the Department of Morphological Sciences and Pathological Anatomy, Faculty of medicine and Biomedical Sciences (University of Garoua) based on the histopathologic criteria from Russo and Russo [29].

### Determination of cytokines levels

The serum levels of interferon gamma (IFN- γ), tumor necrosis factor-alpha (TNF-α), epidermal growth factor (EGF), interleukin-6 (IL-6), interleukin-12 (IL-12) and fractalkine were assessed using magnetic luminex screening assay. This assay was carried out using rats premixed MILIPLEX® Kit (Millipore, Minneapolis, USA) according to the manufacturer’s instructions. In Brief, 96-microplates were incubated on a horizontal shaker with 50 µL/well of serum (previously diluted) and standard cytokines with 50 µL of rat, magnetic, premixed, microparticle cocktail. Afterward, microplates were washed using a magnetic plate separator and incubated with 50 µL/well of rats premixed biotin-antibodies cocktail specific for each cytokine. The obtained antibody-cytokine complexes were revealed using Streptavidin-PE and read in a Luminex MAGPix Analyzer (XMAP Technology, SN, USA) and results were expressed as median fluorescence intensity (MFI). The MFI was converted to cytokine relative concentration via a standard curve specific to each cytokine. The minimum concentration detectable of each cytokine was: 5.2 pg/ml (IFN- γ), 1.9 pg/ml (TNF-α), 0.3 pg/ml (EGF), 0.7 pg/ml (fractaklin), 0.2 pg/ml (IL-6), and 0.4 pg/ml (IL-12).

### Determination of total proteins and oxidative stress parameters

The main markers of oxidative stress and total protein levels were measured in mammary glands (NOR) and tumors. The reduced glutathione (GSH) level was measured following Sehirli et al. [30]. method with some modifications. The activities of catalase and superoxide dismutase (SOD) were assessed by the methods reported by Misra [31] and Sinha [32], respectively. Malondialdehyde (MDA), which is a biomarker of lipid membrane peroxidation was evaluated using Wilbur et *al* [33].) protocol. Total protein level was determined following Gonal et al. [34]. method.

### Breast cancer biomarker CA15-3 assessment

The Cell Biolabs’ Cancer Antigen 15 − 3 ELISA kit was used to measure the breast cancer biomarker CA 15 − 3 level in serum, which is one of the most circulating prognostic biomarkers in clinic regarding breast cancer. This assay measures shed or soluble forms of Mucin-1 (MUC-1) protein, which is a trans-membrane protein consisting of 2 subunits (stable dimer) expressed at the apical plasma membrane of epithelial cells. The detection sensitivity limit of the kit is 4 U/mL CA 15 − 3. The assay was carried out according to manufacturer’s instructions.

### Statistical analysis

The results were expressed as mean ± standard error of the mean (SEM) and analyzed using Graphpad prism 5.0. The ANOVA (analysis of variance) followed by Dunnett’s post-hoc test was used to compare all groups against the negative control group (DMBA). The unpaired student’s t-test was used to compare the differences between the negative control group (DMBA) and the normal control group (NOR). The significance was set at *p* < 0.05.

## Results

### *Phytochemical analysis and in vitro antioxydant potential of A. muricata* Linn extracts

Table 1 summarizes the secondary metabolites and macronutrients contents, and the in vitro antioxidant capacity of *A. muricata* Linn extracts. It was noted that flavonols are the most abundant secondary metabolites with a content of 8.47 ± 2.24 µg ES/g dry weight (DW) in leave (AML) and 5.93 ± 1.4 µg ES/g DW in the fruits (AMF) while tannins was the less represented class with a content of 6.32 ± 1.5 µg EAT/g DW in AML and 12.07 ± 2.1 mg EAT/g DW in AMF.

**Table 1:** Quantitative analysis of selected phytochemical present in *A. muricata* leaves and fruits extracts and in vitro antioxidant potentials

		Content in *A. muricata*	
	Item	AML	AMF
1	Flavonols (µg ES/g DW)	8.47 ± 2.24	5.93 ± 1.40
2	Flavonoids (µg ES/g DW)	27.95 ± 6.35	21.95 ± 1.76
3	Tannins (mg EAT/g DW)	6.32 ± 1.5	12.07 ± 2.1
4	Lipids (g/100 g DW)	29.24 ± 1.10	44.05 ± 2.61*
5	Water content (%)	28.98 ± 0.05	34.54 ± 1.02
6	FRAP	3.46 ± 0.25	2.97 ± 0.25
7	DPPH	40.44 ± 2.25	32.60 ± 1.77

Data are represented as mean ± SEM of triplicates from at least three independent experiments. FRAP, ferric reducing antioxidant power. Significance compared to AML: *p < 0.05. DW = dry weight

Further, *Annona muricata* Linn extracts demonstrated an ability to reduce ion (Fe^2+^) through FRAP assay (3.46 ± 0.25 for AML and 2.97 ± 0.25 for AMF) and to scavenge DPPH free radicals (40.44 ± 2.25 for AML and 32. 60 ± 1.77 for AMF) methods.

### Animal survival and body weight

**Fig. 1:**
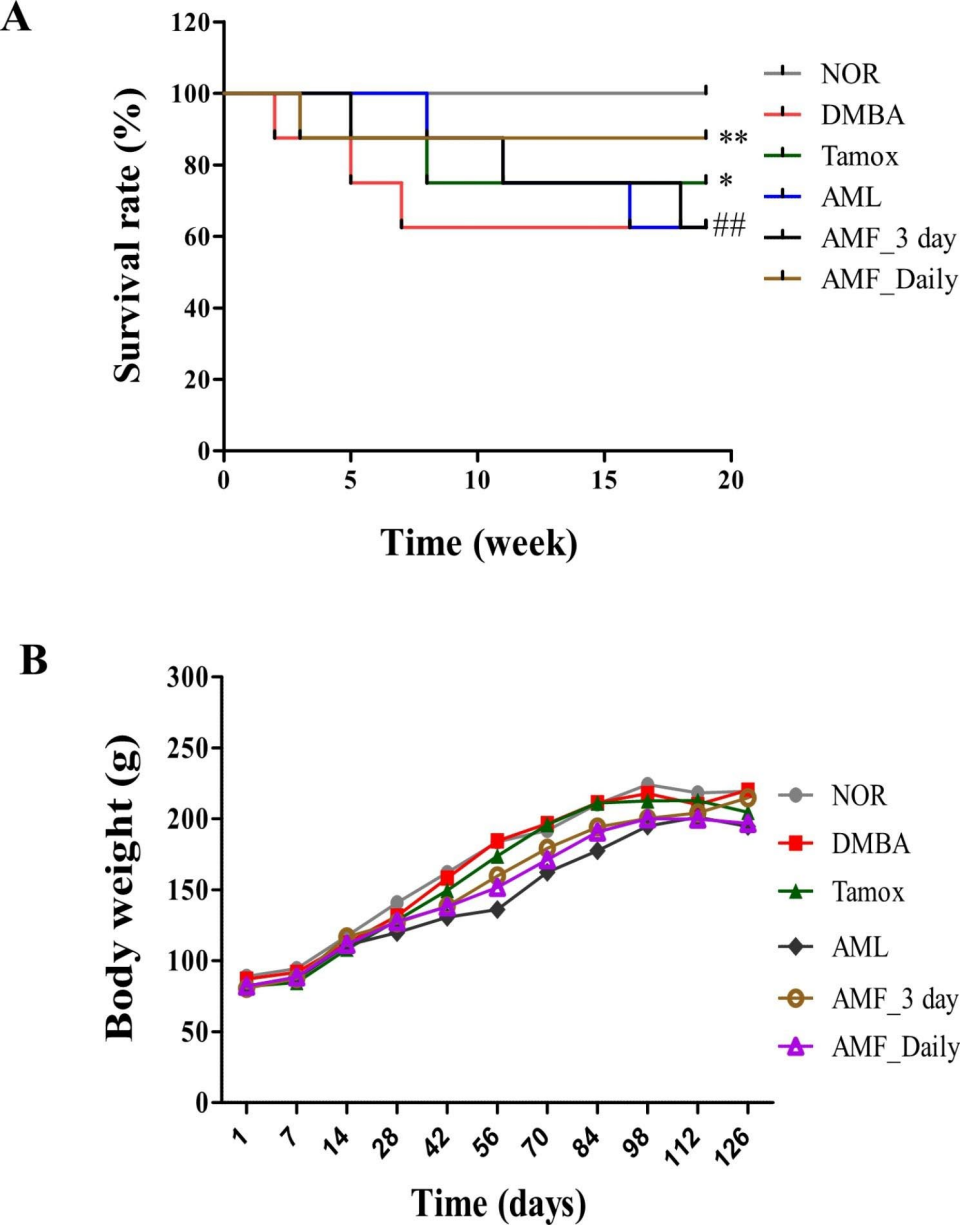
Kaplan Meir curve presenting the survival rate (**A**) and body weight progression (**B**) after 20 weeks of treatment. Normal (NOR) and negative control (DMBA) groups received the vehicle (distilled water); Tamox + DMBA = positive control group, treated with tamoxifen at 3.3 mg/kg BW; AMF + DMBA = rats treated with the ethanolic extract of *A. muricata* Linn leaves at 200 mg/kg BW. AMF = rats treated with *A. muricata* Linn pulp fruit aqueous extract at 200 mg/kg BW at 2 different frequencies (3 times/week and every day). Significance compared to the DMBA group: * *p* < 0.05 and ** *p* < 0.01. Significance compared to the NOR group: ## *p* < 0.01 compared to the NOR group (n = 8)

Figure 1A shows the rate of survival of animals after 20 weeks using the Kaplan Meir curve. No death was noticed in the normal group (NOR), but the DMBA group registered high death rate (3 rats out of 8; 62.5%; (*p* < 0.01), followed by animals treated with AMF 3 days/week and AMF. Rats treated with AMF daily had the lowest incidence of death (1 rat out of 8; 87.53%).

No difference was observed in the body weight progression of animals during the experiment (Fig. 1B).

### Effect of A. muricata Linn on some tumor parameters

Table 2 presents the effects of *A. muricata* on tumor incidence, tumor weight and volume, tumor burden and % inhibition of tumor burden. The normal group had no tumors, while 100% of rats in the negative control group (DMBA) developed mammary tumors. Treatment with tamoxifen reduced tumor incidence to 12.5% compared to the DMBA group. As far as *A. muricata* Linn is concerned, AML reduced the incidence of tumors to 25%, while AMF taken daily reduced tumor incidence to 37.5%. Moreover, AMF 3 days/week reduced tumor incidence to 62.5%.

**Table 2:** Chemo-preventive activity of *A. muricata L.* leaves and fruit extracts after 20 weeks of treatment

	Nber of rats with tumor	Tumor incidence (%)	Tumor weight (mg/kg)	Tumor burden (g)	Inhibition in tumor burden (%)
**NOR**	0/8	0%	-	-	-
**DMBA**	8/8	100%	13.4 ± 1.8	972.9	-
**Tamox**	1/8	12.5%	3.07 ± 0.02***	295.2	69.7
**AML**	2/8	25%	6.9 ± 1.4***	257.7	73.5
**AMF_3 days**	5/8	62.5%	2.2 ± 0.04***	397.9	59.1
**AMF_Daily**	3/8	37.5%	1.3 ± 0.6***	100.8	89.6

Normal (NOR) and negative control (DMBA) groups received distilled water (vehicle); Tamox + DMBA = rats serving as positive control, treated with tamoxifen at 3.3 mg/kg BW; AML + DMBA = rats treated with the ethanolic extract of *A. muricata* leaves at 200 mg/kg BW; AMF = rats treated with *A. muricata* pulp fruit aqueous extract at 200 mg/kg BW at 2 different frequencies (3 times/week and daily). Significance compared to the DMBA group: ^***^p < 0.001 (n = 8)

A significant decrease (*p* < 0.001) in the average relative weight of tumors was observed in rats treated with tamoxifen (from 13.4 ± 1.8 mg/kg in DMBA to 3.07 ± 0.02 mg/kg). The *A. muricata* Linn leaves (AML) reduced the average relative weight of tumors (from 13.4 ± 1.8 mg/kg in DMBA to 6.9 ± 1.4 mg/kg (AML)), but less than the *A. muricata* Linn pulp fruit extract. In fact, the AMF taken 3 days/week (2.2 ± 0.04 mg/kg) or daily (1.3 ± 0.6 mg/kg) was more potent in reducing tumor weight than the AML taken daily.

The highest % inhibition of tumor burden was observed in the groups treated with AML (73.5%) and AMF taken daily (89.60%) (Table 2).

### Effect of A. muricata Linn on tumor volume and CA15-3 level

**Fig. 2:**
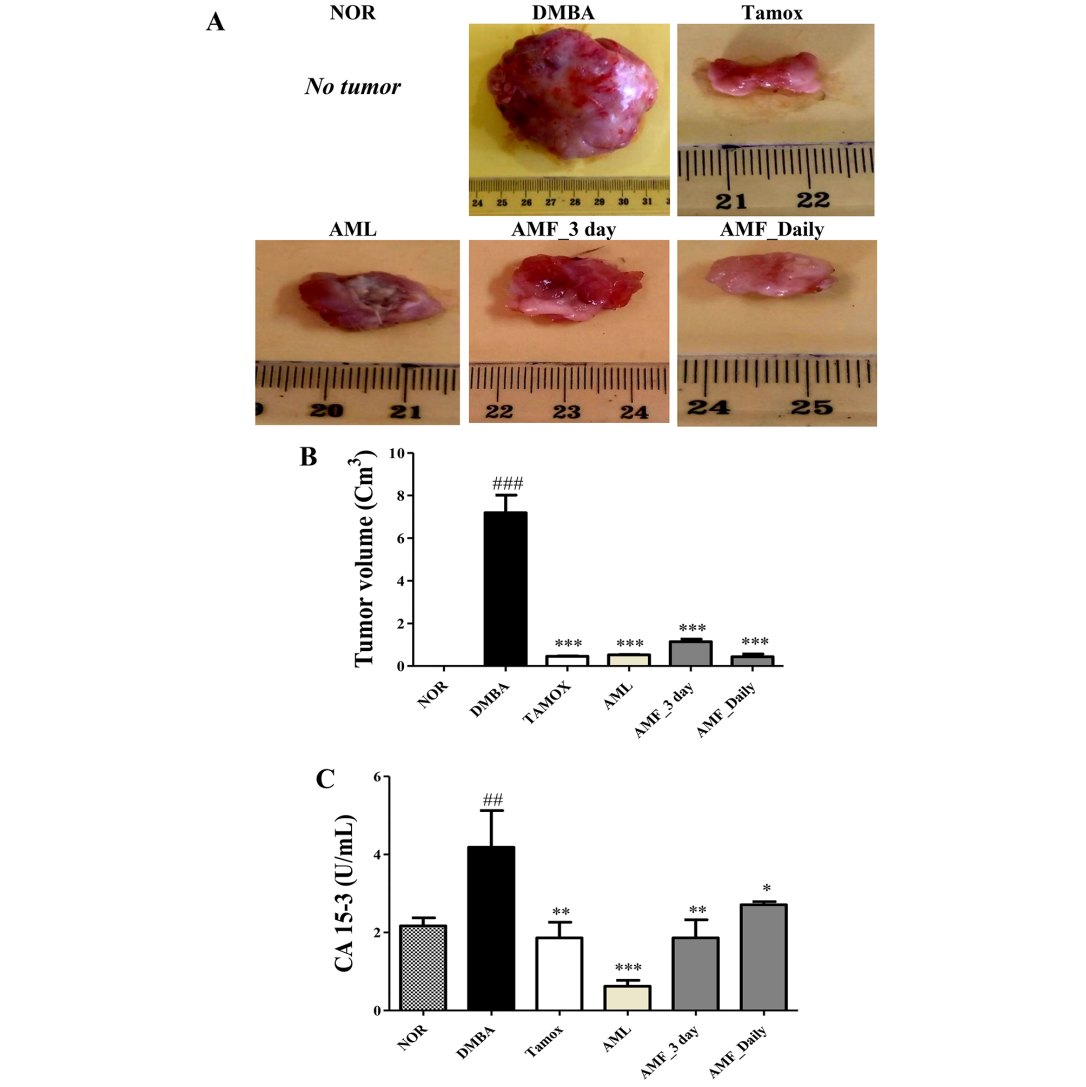
Effects of *A. muricata* Linn leaves extract and juices on tumor morphology (**A**) tumor volume (**B**) and CA15-3 level (**C**). Normal (NOR) and negative control (DMBA) groups received the vehicle (distilled water); Tamox + DMBA = positive control group, treated with tamoxifen at 3.3 mg/kg BW; AMF + DMBA = rats treated with the ethanolic extract of *A. muricata* Linn leaves at 200 mg/kg BW. AMF = rats treated with *A. muricata* Linn pulp fruit aqueous extract at 200 mg/kg BW at 2 different frequencies (3 times/week and every day). Significance compared to the DMBA group: * *p* < 0.05; ** *p* < 0.01 and *** *p* < 0.001. Significance compared to the NOR group: ##*p* < 0.01 and ### *p* < 0.001 compared to the NOR group (n = 8)

Figure 2 shows that rats exposed solely to DMBA had the largest tumors volume compared to the other groups. Treatment with the standard drug tamoxifen significantly (*p* < 0.001) reduced tumor volume (from 7.2 ± 0.8 cm^3^ in DMBA to 0.45 ± 0.0015 cm^3^) and CA15-3 level (from 4.18 ± 0.93 U/ml in DMBA to 1.86 ± 0.38 U/ml). *A. muricata* Linn leaves and pulp fruit extract at all regimens (3 days/week or daily) also significantly (*p* < 0.001) reduced tumor volume (from 7.8 ± 0.8 cm^3^ in DMBA to 1.13 ± 0.11 cm^3^ in AMF taken 3 day/week) and CA15-3 level (from 4.18 ± 0.93 U/mL in DMBA to 1.86 ± 0.46 U/ml in AMF taken 3 day/week). This later effect was however more pronounced with AML than in AMF taken daily.

### Histopathology of breast tumors

**Fig. 3:**
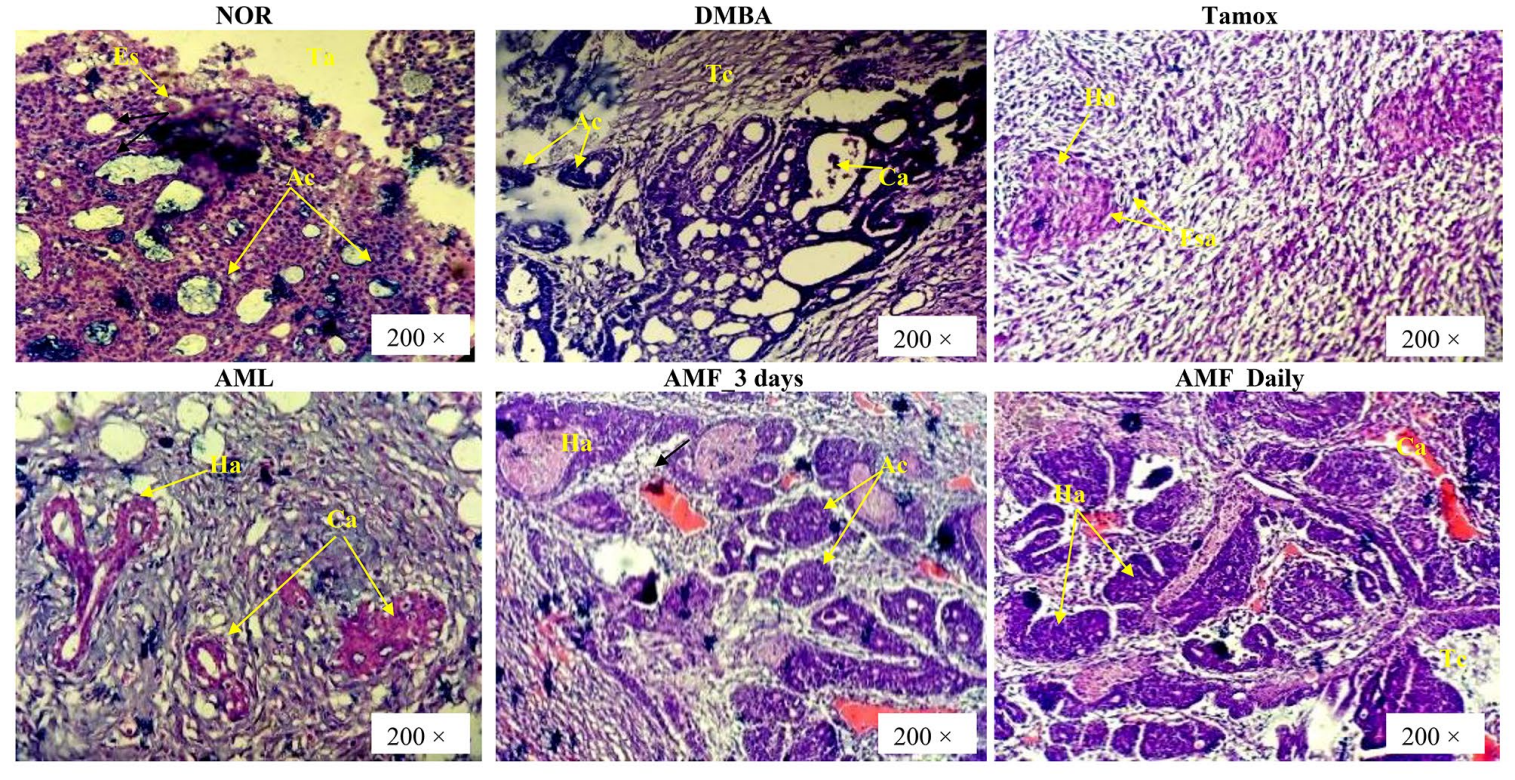
Histopathology of mammary glands and mammary tumors. Normal (NOR) and negative control (DMBA) groups received the vehicle (distilled water); Tamox + DMBA = positive control group, treated with tamoxifen at 3.3 mg/kg BW; AMF + DMBA = rats treated with the ethanolic extract of *A. muricata* Linn leaves at 200 mg/kg BW. AMF = rats treated with *A. muricata* Linn pulp fruit aqueous extract at 200 mg/kg BW at 2 different frequencies (3 times/week and every day). Ac = Acinus ; At = Adipose tissue ; Ct = Conjonctive tissue ; Ca = Carcinoma ; Ly = Lymphocyte ; Ha = Atypical Hyperplasia; Fsa = Fibrosarcoma ; Es = Eosinophile secretion ; Co = Codemonecrose

Photomicrographs (Fig. 3) of the mammary glands from normal rats (NOR) show normal mammary parenchyma with acini presenting mononuclear cells surrounded by adipocytes. Sections of breast tumors from the DMBA group showed high Scarff-Bloom-Richardson grade (SBR III) cribriform ductal carcinoma with low lymphocyte infiltration (< 5%) and ~ 30% comedonecrosis (Table 3). Breast tumors from rats treated with tamoxifen had predominantly low-grade (SBR I) fibrosarcoma without lymphocyte infiltration and 0% comedonecrosis. Rats treated with *A. muricata* Linn leaves extract (AML) presented cribriform ductal carcinoma of grade SBR II, with 30% lymphocyte infiltration, while those treated with *A. muricata* Linn pulp fruit extract showed predominantly cribriform ductal carcinomas of SBR grade II in both regimens. It is noteworthy to mention that only rats receiving *A. muricata* Linn leaves and fruit extract treated daily had lymphocyte infiltration (30% for AML and 20% for AMF).

**Table 3:** Histological types of tumors obtained following DMBA exposition

	Histological type	SBR grade	Lymphocyte infiltration (%)	Nécrosis & comedonecrosis
**DMBA**	Cribriform ductal carcinoma	III	< 5%	30%
**Tamox**	Fibrosarcoma	I	0%	0%
**AML**	Cribriform ductal carcinoma	II	30%	20%
**AMF-3 day**	Cribriform ductal carcinoma	II	0%	20%
**AMF_ Daily**	Cribriform ductal carcinoma	II	20%	0%

Normal (NOR) and negative control (DMBA) groups received distilled water (vehicle); Tamox + DMBA = rats serving as positive control, treated with tamoxifen at 3.3 mg/kg BW; AML + DMBA = rats treated with the ethanolic extract of *A. muricata* leaves at 200 mg/kg BW; AMF = rats treated with *A. muricata* pulp fruit aqueous extract at 200 mg/kg BW for 2 different frequencies (3 times/week and every day)

### Effects of A. muricata Linn on some inflammatory cytokines

**Fig. 4:**
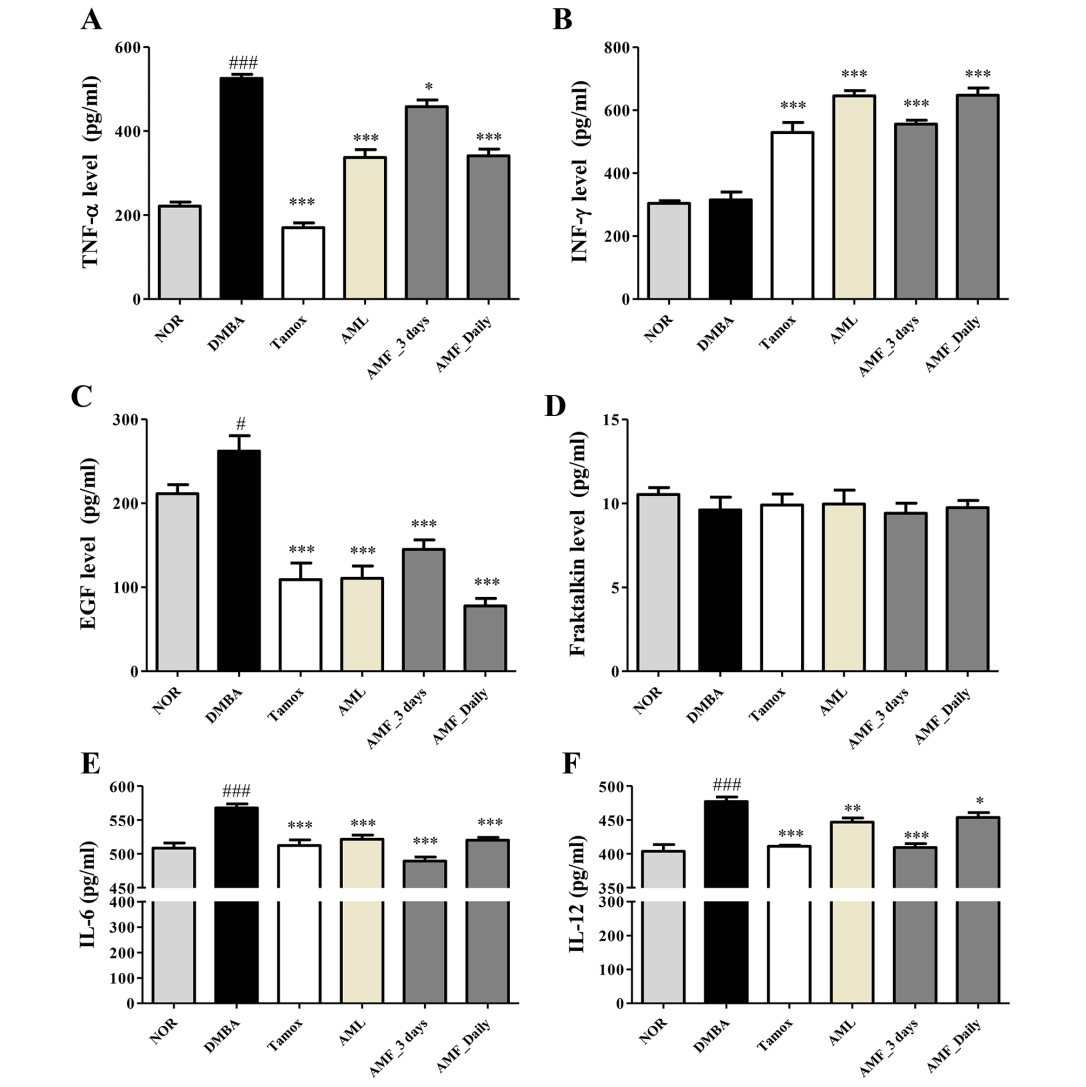
Effects of *A. muricata* Linn leaves and fruit extracts on some cytokine levels. Normal (NOR) and negative control (DMBA) groups received the vehicle (distilled water); Tamox + DMBA = positive control group, treated with tamoxifen at 3.3 mg/kg BW; AML + DMBA = rats treated with the ethanolic extract of *A. muricata* Linn leaves at 200 mg/kg BW. AMF = rats treated with *A. muricata* Linn pulp fruit aqueous extract at 200 mg/kg BW at 2 different frequencies (3 times/week and every day). Significance compared to the DMBA group: * *p* < 0.05; ** *p* < 0.01 and *** *p* < 0.001. Significance compared to the NOR group: #*p* < 0.05 ### *p* < 0.001 compared to the NOR group (n = 8)

Figure 4 depicts the effects of different treatments on cytokine levels in serum after 20 weeks of experiments. DMBA induced a significant increment in the levels of TNF-α (*p* < 0.001), IL-6 (*p* < 0.001), IL-12 (*p* < 0.001) and EGF (*p* < 0.05), but did not significantly change the levels of IFN-γ and fractalkine compared to the normal control group. Tamoxifen treatment significantly (*p* < 0.001) reduced the levels of TNF-α, IL-6, IL-12 and EGF, while they significantly increased the levels of IFN γ compared to the DMBA group. All groups treated with *A. muricata* Linn leaves extract as well as pulp fruit extract significantly (*p* < 0.001) decreased the serum levels of TNF-α and EGF compared to DMBA with the exception of AMF taken 3 days/week which inhibited significantly (*p* < 0.05). No significant change was observed in the level of fractalkine after different treatments. In addition, *A. muricata* Linn leaves and fruit extract at all the regimen significantly (*p* < 0.001) increased the level of IFN-γ and decreased the levels of IL-6 and IL-12 as compared to the DMBA group just like the tamoxifen.

### Effect of A. muricata Linn on some parameters of oxidative stress, ALT and creatinine

Table 4 depicts the results obtained regarding the effects of *A. muricata* Linn leaves and fruit extracts on some oxidative stress markers after 20 weeks of experiment. There was a significant increase (*p* < 0.05) in total protein level in the DMBA group compared to the normal group. The group receiving the standard drug (Tamox) as well as *A. muricata* Linn leaves and fruit extracts reversed the increase in total proteins level induced by the DMBA, with optimal effect with AMF taken daily (from 0.9 ± 0.01 to 0.1 ± 0,003 g/l).

**Table 4:** Effect of *A. muricata* on some parameters of oxidative stress, alanine transaminase (ALT) activity and creatinine level

	NOR	DMBA	TAMOX	AML	AMF_3 days	AMF_daily
**Proteins** (g/l)	0.3 ± 0.01	0.9 ± 0.01#	0.7 ± 0.02	0.1 ± 0.02**	0.6 ± 0.01	0.1 ± 0.003**
**Catalase**(mM of H_2_O_2_/min/mg P)	2.36 ± 0.4	4.6 ± 0.25	3.7 ± 0.7	8.3 ± 1.5	8.7 ± 0.62*	9.8 ± 0.36**
**MDA**(mM/mg P)	9.3 ± 0.9	40.2 ± 1.04##	15.1 ± 1.02*	39.3 ± 4.2	25.75 ± 5.03	14.5 ± 6.8*
**GSH**(mM/mg P)	25.3 ± 2.8	11.3 ± 1.6##	25.8 ± 2.4 **	22.1 ± 1.5*	17.1 ± 2.2	26.4 ± 1.2**
**SOD**(unity/mg P)	1.3 ± 0.2	0.61 ± 0.3	1.71 ± 0.2*	1.3 ± 0.2*	1.0 ± 0.01	1.3 ± 0.3*
**ALAT**(U/I)	230.6 ± 35.6	362.3 ± 11.6##	259 ± 36.9*	259 ± 25.8*	35.6 ± 9.9***	113.2 ± 22.4**
**Créatinine**(g/l)	0.33 ± 0.1	0.75 ± 0.2#	0.4 ± 0.1	0.43 ± 0.06	0.38 ± 0.02	0.32 ± 0.02*

Normal (NOR) and negative control (DMBA) groups received distilled water (vehicle); Tamox + DMBA = rats serving as positive control, treated with tamoxifen at 3.3 mg/kg BW; AML + DMBA = rats treated with the ethanolic extract of *A. muricata* leaves at 200 mg/kg BW. AMF = rats treated with *A. muricata* pulp fruit aqueous extract at 200 mg/kg BW at 2 different frequencies (3 times/week and every day). Significance compared to the NOR group: #*p* < 0.05, ##*p* < 0.01. Significance compared to the DMBA group: *p < 0.05, **p < 0.01, ***p < 0.001. mg P = mg of proteins

Concerning catalase activity, there was a non-significant increase of catalase activity in the negative control group compared to the normal group. Rats treated with AMF 3 day/week and daily significantly (at least *p* < 0.05) increased catalase activity.

There was a significant (*p* < 0.01) increase in MDA levels in the DMBA group compared to the normal group. Tamox, AMF taken daily significantly (*p* < 0.05) prevented the lipid membrane peroxidation induced by DMBA. AML and AMF taken 3 days/week presented a non-significant decrease of this parameter.

With regards to SOD activity, the DMBA group showed decreased activity compared to normal rats. All treated groups with the exception of AMF taken 3 days/week significantly (*p* < 0.05) increased SOD activity.

DMBA group significantly (*p* < 0.01) reduced the level of GSH as compared to normal rats. All treated groups increased GSH levels although a significant (*p* < 0.05) increase was observed only with AML, AMF taken daily.

DMBA-rats showed a significant (*p* < 0.01) increment in ALT activity (from 230.6 ± 35.6 in normal rats to 362.3 ± 11.60 in DMBA) and creatinine level (from 0.33 ± 0.1 in normal rats to 0.75 ± 0.20 in DMBA) compared to normal rats. All treatments diminished the activity of ALT and creatinine levels. The decrease in ALT activity was statistically significant in all groups, but the decrease in creatinine level was only significant (*p* < 0.05) in the AMF (daily) group.

### Effect of A. muricata Linn on relative organ weights and hematological parameters

Table 5 shows that DMBA induced a significant increase in liver (*p* < 0.01) and spleen (*p* < 0.05) wet weights and a significant decrease in thymus (*p* < 0.001) and femur (*p* < 0.05) wet weights, while it did not change the kidneys wet weight. All the treated groups significantly (*p* < 0.001) prevented the reduction of thymus weight apart from AMF taken 3 days/week (non-significant). As far as the liver weight is concerned, all treated groups prevented its increment, although non-significant with AMF taken 3 days/week. All treated groups significantly (*p* < 0.001) prevented the increment in spleen wet weight compared to DMBA rats.

**Table 5:** Effect of *A. muricata* on the relative weight of some organs and hematological parameters

Organs (mg/kg)	NOR	DMBA	TAMOX	AML	AMF_3 days	AMF_Daily
Thymus	1396.7 ± 40.3	768 ± 40.3###	1423.7 ± 20.9***	439.3 ± 27.1***	826.5 ± 54.3	1116.1 ± 35.1***
Liver	30,381 ± 624.7	35214.6 ± 1331.6##	30922.6 ± 235.4*	30794.4 ± 908.1**	33716.5 ± 1684.2	30970.6 ± 396.6*
Kidneys	5618 ± 113.6	5780.8 ± 322.3	5902.1 ± 83.6	5767 ± 136.3	5888.7 ± 115.6	5506.3 ± 80.9
Femur	2569.7 ± 84.7	2257 ± 106#	3044.2 ± 41.1***	3217.7 ± 92.5***	2960.1 ± 47.2	2673.1 ± 75.4**
Spleen	6000 ± 85.5	7127.4 ± 710.6#	4335.3 ± 44.5***	3413.2 ± 69.1***	4263.6 ± 180.1***	4148.1 ± 120.8***
**Hematology**						
WBC (×10^3^/µl)	12.5 ± 0.54	15.42 ± 0.51##	12.8 ± 0.7	9.9 ± 1	351.2 ± 128.1***	6 ± 1.1
Lymphocytes (%)	69.8 ± 5	66 ± 0.4	79.7 ± 2.5*	73.7 ± 3.9	79.6 ± 3.4	70.2 ± 3.3
Monocytes (%)	5.2 ± 0.5	6.32 ± 0.9	4.6 ± 0.9	13.3 ± 4.2	24.4 ± 2.4***	15.6 ± 0.6**
Granulocytes (%)	18.4 ± 1.5	27.62 ± 1.34##	16.8 ± 1.4***	9.9 ± 1.3***	11.2 ± 1.2***	19.5 ± 0.4***
RBC (×10^3^/µl)	8 ± 0.5	5.58 ± 0.76#	7.8 ± 0.4*	6.6 ± 0.4	6.2 ± 0.2	7.1 ± 0.4*

Normal (NOR) and negative control (DMBA) groups received distilled water (vehicle); Tamox + DMBA = rats serving as positive control, treated with tamoxifen at 3.3 mg/kg BW; AML + DMBA = rats treated with the ethanolic extract of *A. muricata* leaves at 200 mg/kg BW; AMF = rats treated with *A. muricata* pulp fruit extract at 200 mg/kg BW at 2 different frequencies (3 times/week and every day). Significance compared to the NOR group: #*p* < 0.05, ##*p* < 0.01, ###*p* < 0.001. Significance compared to the DMBA group: *p < 0.05, **p < 0.01, ***p < 0.001

Hematological analysis showed that white blood cell count increased in the DMBA group as compared to normal group (from 12.5 ± 0.54 × 10^3^/µl in normal rats to 15.42 ± 0.51 × 10^3^/µl in DMBA). The subpopulation mainly affected was granulocytes which also significantly increased (from 18.4 ± 1.50% in normal rats to 27.62 ± 1.34% in DMBA). All treatments significantly (*p* < 0.001) prevented the increase in granulocytes. In the same scheme, all treatments increased the % of lymphocytes although significant solely with Tamox (*p* < 0.05) group. DMBA induced a non-significant increase in monocytes subpopulation. Only AMF at both regimens induced a significant increase in the monocytes.

DMBA treatment significantly (*p* < 0.05) reduced red blood cell count (from 8 ± 0.46 × 10^3^/µl in normal rats to 5.58 ± 0.76 × 10^3^/µl in DMBA). The tamoxifen prevented significantly (*p* < 0.05) this decrement. As far as *A. muricata* is concerned, only AMF taken daily (from 5.58 ± 0.76 × 10^3^/µl in DMBA rats to 7.10 ± 0.43 × 10^3^/µl) prevented the DMBA-induced decrease in RBC count compared to DMBA.

### Effect of A. muricata Linn on histoarchitecture of some organs

**Fig. 5:**
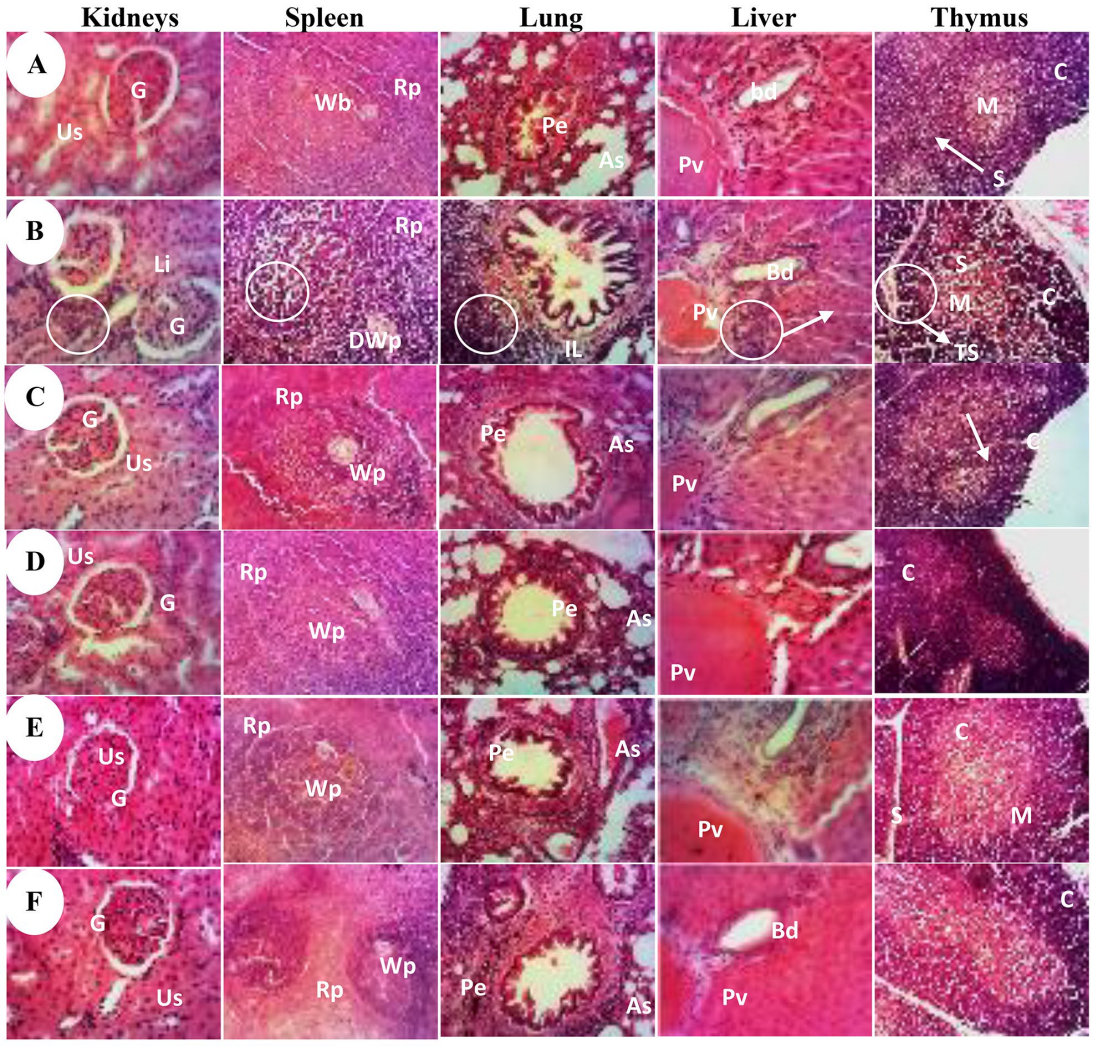
Effects of *A. muricata* Linn leaves extract and juices on the microarchitecture of kidney (×200), spleen (×100), lung (×100), liver (×200) and thymus (×400) following a H&E staining. Normal (**A**) and negative control (**B**) groups received the vehicle (distilled water); C = positive control group, treated with tamoxifen at 3.3 mg/kg BW; D = rats treated with the ethanolic extract of *A. muricata* Linn leaves at 200 mg/kg BW. E & F= ### *p* < 0.001. Us = urinary space, G = glomerulus, LI = leukocyte infiltration, Wp = white pulp, Rp = red pulp, DWp = Disorganization of white pulp, Pe = pulmonary epithelium, As = alveolar sac, Pv = portal vein, Bd = bile duct, M = medullary layer, S = Interlobular septum, C = cortex, TS = Thickening of the interlobular septum

Figure 5 shows the effects of different treatments on kidneys, spleen, liver, lung and thymus microarchitectures. The normal group had kidneys with urinary space and well-differentiated glomerulus, their liver had normal parenchyma with mononuclear hepatocytes. The histoarchitecture of the lungs was normal with normal alveolar bags, while the spleen had well-differentiated white and red pulp in normal parenchyma. Sections from DMBA rats presented leukocytes infiltration into the liver, lungs and kidneys and a disorganization of the white pulp of the spleen. DMBA also induced a decrease in the density of cortical lymphocytes and a thickening of the interlobular septum in the thymus compared to the normal control group. Tamoxifen and *A. muricata* Linn treatments prevented the decrease in cortical lymphocyte density in the thymus, the leukocyte infiltration in kidneys, hepatic cells and lungs as well as the disorganization of the white pulp of the spleen.

### Comparative anticancer effects of Annona muricata L fruits and Leaves

Table 6 Summarizes the overall results obtained with *A. muricata* L leaves and fruits both taken daily. The bold highlights the most active part according to the assessed parameter. The two parts have close activities but taken all in all, the fruit aqueous extract is slightly more potent than the ethanolic leaves extract.

**Table 6:** Comparative anticancer effects of *Annona muricata* L fruits and leaves on different parameters in DMBA-induced breast cancer in rats

			*Annona muricata* L (200 mg/kg BW daily)	
N°	Parameters	NC	Leaves	Fruits
1	Flavonoid (µg ES/g DW)	-	**27.95 ± 6.35**	21.95 ± 1.76
2	Tannin (mg EAT/g DW)	-	6.32 ± 1.5	**12.07 ± 2.1**
3	Antioxidant FRAP	-	**3.46 ± 0.25**	2.97 ± 0.25
4	Antioxidant DPPH	-	**40.44 ± 2.25**	32.60 ± 1.77
5	Rate of death	62.5%	75%	**87.53%**
6	Incidence of tumors	100%	**25%**	37.5%
7	Tumor weight (mg/kg)	13.4 ± 1.8	6.9 ± 1.4	**1.3 ± 0.6**
8	% inhibition tumor burden	-	73.5%	**89.60%**
9	Tumor volume (cm^3^)	7.2 ± 0.8	0.52 ± 0.01	**0.43 ± 0.02**
10	CA15-3 level (U/ml)	4.18 ± 0.93	**0.62 ± 0.15**	2.7 ± 0.07
11	Histopathology tumors	SBR III	**SBR II + 30% TIL**	**SBR II + 20% TIL**
12	TNF-α (pg/mL)	527.61 ± 13.09	348.6 ± 24.48	**341.4 ± 20.61**
13	IFN γ (pg/mL)	310.21 ± 31.94	641.66 ± 21.61	**674.66 ± 29.69**
14	EGF (pg/mL)	262.2 ± 23.64	110.6 ± 19.06	**77.74 ± 11.71**
15	IL-6 (pg/mL)	567.59 ± 6.99	521.40 ± 7.15	**519.96 ± 4.88**
16	IL-12 (pg/mL)	477.06 ± 7.89	**446.80 ± 7.16**	453.53 ± 8.25
17	Proteins in tumor (g/l)	0.9 ± 0.01	**0.1 ± 0.02**	**0.1 ± 0.00**
18	CAT (mM of H_2_O_2_/min/mg P)	4.6 ± 0.25	8.3 ± 1.5	**9.8 ± 0.36**
19	MDA(mM/mg P)	40.2 ± 1.04	39.3 ± 4.2	**14.5 ± 6.8**
20	GSH (mM/mg P)	11.3 ± 1.6	22.1 ± 1.5	**26.4 ± 1.2**
21	SOD (unity/mg P)	0.61 ± 0.3	**1.3 ± 0.2**	**1.3 ± 0.3**
22	ALAT (U/I)	362.3 ± 11.6	259 ± 25.8	**113.2 ± 22.4**
23	Créatinine (g/l)	0.75 ± 0.2	0.43 ± 0.06	**0.32 ± 0.02**
24	WBC (×10^3^/µl)	15.42 ± 0.51	9.9 ± 1	**6 ± 1.1**
25	RBC (×10^3^/µl)	5.58 ± 0.76	6.6 ± 0.4	**7.1 ± 0.4**
26	Prevent the DMBA-induced damage		**++**	**++**

Bold = more efficient; + = protective effect; - = no applicable. DW = dry weight; P = protein, NC = negative control; TIL = Tumor’s infiltrating lymphocytes

## Discussion

Breast cancer is the most common type of cancer in women worldwide including Cameroon [5]. They are available treatments, with many drawbacks and their high-cost limit their usage particularly in developing countries, where there is a high mortality rate due to several other challenges such as poor health infrastructure and delayed health-seeking behavior [9, 35]. Therefore, many patients from these regions rely on traditional medicine, which is accessible and has fewer side effects. In fact, according to the World Health Organization, 80% of the populations in Africa and in some Asian countries still use plant preparations to treat their illnesses, including cancer [10]. Thus, research on natural antitumor substances is a current need and one of the main strategies is to study local natural substances on the basis of their traditional uses [11]. Thus, the present work was carried out to better understand the anticancer potential of *A. muricata* Linn juices.

The polycyclic aromatic hydrocarbon DMBA has been reported to induce breast cancer in female rats during hormonal transition (~ 60 days), when their mammary terminal ducts undergo active proliferation [36]. In line with the above, 100% of rats exposed solely to DMBA, developed mammary tumors in this study. Of note, DMBA-induced breast cancer in rats is a popular estrogen-dependent cancer model which, develops from ductal epithelial cells and closely resembles cancer of women biochemically, immunologically and histologically [29]. The histopathology of mammary gland showed that DMBA induced a cribriform ductal carcinoma of SBR grade III with low lymphocyte infiltration. Also, the biomarker CA 15 − 3 level increased significantly in the DMBA’ serum group. Both CA 15 − 3 and the Scarff-Bloom-Richardson (SBR) grade are important prognostic factors in breast cancer, which are usually associated with high cell proliferation rate [37]. The above also justifies the great use of this animal model, since 70% of breast cancer in women is mainly ductal carcinoma. In Cameroon just like in many developing countries, breast cancer is most often diagnosed at a high grade due to the multiple itineraries of the patients. With this cancer model, the initiator DMBA induces irreversible changes in cellular genes and endogenous estrogens promotes proliferation of initiated cells leading to abnormal growth and further mutations which can progress in other tissues [38].

In this study, extracts from *A. muricata* Linn (leaves and pulp fruit) as well as the positive control (tamoxifen) significantly reduced the death incidence, tumor incidence, tumor volume and weight, total protein levels and CA 15 − 3 levels compared to the DMBA group. This suggests a protective effect of tested substances on breast cancerogenesis. Cancer is usually characterized by increased cellular protein levels, as observed in this study. Mucin-1 protein, a transmembrane protein on the surface of plasma membranes of epithelial cells is a biomarker of cancer. It is overexpressed in breast cancer which gives it a prognostic value [39]. The increased expression of Mucin-1 in this study was in accordance with tumor growth and different treatments decreased its level. The selective estrogen receptor modulator (tamoxifen) significantly reversed cancerogenesis as it could prevent the multiplication of cancer cells by competing with endogenous estrogens for ERs binding in the mammary glands [40]. These effects are in line with many studies, which showed the protective effect of tamoxifen in DMBA-induced breast cancer in rats [41, 42].

Many studies have reported the inhibitory potential of *Annona muricata* Linn on breast cancer induced in female rats. A previous study reported that *Annona muricata* Linn leaves (300 mg/kg) were able to inhibit breast cancer ductal with infiltrative grade II, improve histological changes and reduce the proliferative indexes induced by DMBA [43]. In another study, DNA smears obtained from agarose gel electrophoresis suggested possible DMBA-induced damage which was significantly prevented by *A. muricata* Linn leaf extract and photomicrographs from histological assay revealed the presence of DMBA-induced lobular alveolar hyperplasia, adenomatoid hyperplasia, fibro adipose stroma, and proliferating sebaceous gland in sections of the breast tissues of treated mice thus confirming its chemoprotective effect [16]. Therefore, *A. muricata* Linn leaves extract is a potential anticancer agent. Its chemopreventive effects are due to its bioactive metabolites which may cause arrest of the cell cycle at the G1 phase, promotes apoptosis through Bax and caspase-3-related pathway, antioxidant pathway and downregulating estrogen receptors [13, 44]. Flavonol triglycosides (FTGs), alkaloids (ALKs), phenolics (PLs), megastigmanes (MGs), cyclopeptides (CPs), Annonaceous acetogenins (AGEs) and essential oils, are the different phytoconstituents reported in *A. muricata* Linn. They exert their cytotoxicity either through inhibition of ATP synthesis by blocking the mitochondrial complexes, or by promoting apoptosis through upregulation of Bax and downregulation of Bcl-2 and oncogenes [45].

We found in this study that the *A. muricata* pulp fruit aqueous extract reduced the incidence, the volume and the CA15-3 level, which could be attributed to *A. muricata* Linn bioactive molecules against carcinogenesis, in particular polysaccharides that are effectively involved in the processes of inhibiting the growth of tumor cells and promoting the process of programmed cell death (apoptosis) [46]. However, tumor reduction was more marked in animals receiving the *A. muricata* Linn pulp fruit extract daily as compared to those receiving AML. Referring to the mode of consumption of *A. muricata* fruit extract by the population, two frequencies of administration were studied: 3 days a week and every day. The daily administration showed better chemo-preventive effects.

To gain more insight on the effect of *A. muricata* Linn leaves and pulp fruit extract, the antioxidant status of animals was evaluated. In fact, it is well established that DMBA-induced mammary carcinoma in rats partly acts through the generation of reactive oxygen species (ROS), which in turn damage DNA [47]. In accordance with the decrease in tumor incidence, tumor volume and weight as well as total proteins and CA 15 − 3 levels, *A. muricata* Linn fruit and leaves extracts exhibited an antioxidant effect. This was confirmed by the increase in GSH levels, SOD and catalase activities and the decrease in MDA levels in treated groups compared to the DMBA group. Of note, cytoplasmic SOD and catalase are ubiquitous enzymes that protect cells from free radicals produced during carcinogenic metabolism [47]. Their significant increase following *A. muricata* Linn treatment suggests its free radical scavenging ability, which is in line with our in vitro results and also with many reports in the literature [47]. In addition, GSH, which is a first line of defense against ROS was increased and MDA (lipid peroxidation marker) was decreased as compared to DMBA. The overall support the antioxidant effect of *A. muricata* Linn extract, which in turn accounts for its anticancer effects. However, the protective effect of *A. muricata* Linn was more marked in rats receiving pulp fruit aqueous extract.

Inflammation plays an important role in the initiation, promotion, angiogenesis and metastasis of tumors [48]. In order to elucidate the anti-neoplasic, immunological mechanisms attributed to *Annona muricata* Linn, several cytokines levels were measured among which IL6; IL-12; TNF-alpha, EGF and fraktaklin. The pro-inflammatory mediators such as TNF-α and IL-6 are mainly involved in cancer-related inflammation and inhibition of these cytokines could protect against chemically induced breast tumors [47]. *A. muricata* Linn significantly (*p* < 0.001) reduced the serum levels of TNF-α (an inflammatory agent highly expressed in breast carcinomas) and IL-6 (a regulator of immune response during infection, which promote tumor development during the later stages of cancer) [47, 49]. This decrement would inhibit tumor proliferation and prevent the secretion of several pro-inflammatory cytokines such as interleukin 17 (not measured in this study). IL-6 is an inflammatory cytokine known to regulate many cancers among which breast cancer and studies have shown that up-regulation of IL-6 lead to cancer cells resistance to chemotherapeutic drugs [50]. Noteworthy, in this study, a significant decrease of IL-6 was observed in treated groups with *Annona muricata* Linn. On the other hand, IL-12 regulates the formation of new blood vessels into growing tumors [51]. Interestingly, a significant decrease was observed in treated groups with *Annona muricata* Linn in comparison to DMBA. TNF-α is also an inflammatory cytokine, a necrotic and tumor-promoting factor in the tumor microenvironment, highly expressed in breast adenocarcinomas. A decrease in these inflammatory cytokines suggest *Annona muricata* Linn is rich in anti-inflammatory compounds such as Annonacin, Annomuricin E, Muricoreacin, quecertin and Murihexocin C [52]. These compounds such as curcumin, could induce a down regulation of these cytokines by regulating the NF-κB pathways thus inhibiting NF-kB cytokine mediated activation such as IL-6 and IL-12 which passes through its activation on AMPK, disturbing the NF-κB pathway by acting on p65 and as a consequence, downregulating transcription of cytokine genes [53]. Also, this reduction in pro-inflammatory cytokine secretion could be due to the ability of these compounds to suppress LPS, LPS-induced NF-κB and MAPK pathways and the levels of p-JNK, p-ERK1/2 and p-p38 in MAPK pathway [54]. The high level of INF-γ usually correlates with a growth of the tumor and its reduction is a good prognosis in cancer management [55]. It was increased after *A. muricata* Linn treatment in this study.

The survival rate of rats in the DMBA group was low (~ 60%) compared to other groups, denoting higher morbidity which is in agreement with the highest breast tumor incidence, tumor burden and volume. This could be explained by the immunotoxicity of this carcinogen against lymphoid organs [56]. In addition, DMBA induced a significant increase in liver and spleen wet weights and a significant decrease in thymus and femur wet weights, while it did not change the wet weights of the kidneys. Change in relative organ weights is an indicator of the potential harmful effects of toxic substances [57]. The significant increase in liver wet weight following DMBA administration correlates with the increase in ALT activity, a biomarker of hepatic function. Although it did not change the kidneys wet weight, DMBA significantly increased creatinine level, in line with the disorganization of the kidney parenchyma and leukocytes infiltration observed via histopathology analysis. *A. muricata* Linn leave and fruit extracts prevented the increase in liver and kidneys weights, ALT activity as well as creatinine levels, suggesting a protective effect in the liver. Further, the significant decrease in red blood cell count and significant increase in white blood cell count in the DMBA group compared to the normal group established the hematotoxicity of DMBA. Indeed, the hematopoietic system is a very sensitive target to xenobiotics and thus constitutes a vital indicator of the physiological and pathophysiological status of an organism [46]. *A. muricata* Linn protected blood cells against DMBA-induced hemolysis, thus strengthening its safety in vivo.

## Conclusion

In this study, 100% of rats exposed to DMBA developed cribriform ductal carcinoma of SBR grade III. *A. muricata* Linn extracts (leaves and fruit) and tamoxifen significantly reduced the incidence of death, tumor incidence, volume and weight of tumors, total protein levels in tumor and the levels of CA 15 − 3 compared to the DMBA group. *A. muricata* Linn exhibited antioxidant activity, evidenced by an increase in GSH levels, SOD and catalase activities and decreased MDA levels compared to DMBA. The pro-inflammatory mediators such TNF-α and IL-6 as well as the INF-γ level were reduced following *A. muricata* Linn treatment. Taken altogether, these results confirm the anti-breast tumor effect of *A. muricata* Linn. However, the pulp fruit aqueous extract better protected against breast cancer than the leaves ethanolic extract, this must be taken into consideration during its consumption for the prevention breast cancer.

## Data Availability

The data and materials used in this study are available upon request from the corresponding author (stephanezingue@gmail.com).

## Abbreviations

AMF: *A. muricata* Linn pulp fruit extract
AML: *A. muricata* Linn leaves
BW: Body weight
BSA: Bovine Serum Albumin
CA 15 − 3: Cancer antigen 15 − 3
DMBA: 7,12 dimethylbenz(a)anthracene
EDTA: Ethylenediaminetetraacetic
EGF: Epidermal growth factor
ELISA: Enzyme-linked immunosorbent assay
ER: Estrogen receptor
ERK: Extracellular regulated kinase
Hb: Hemoglobin
IL-6: Interleukin-6
IL-12: Interleukin-12
IFN-γ: Interferon gamma
LPS: Lipopolysaccharide
MDA: Malondialdehyde
MAPK: Mitogen activated protein kinase
NHC: National Herbarium of Cameroon
NOR: Normal control
PAHs: Polycyclic aromatic hydrocarbon
RBC: Red blood cell
ROS: Reactive oxygen species
Scarff-Bloom-Richardson (SBR): SEM, standard error of mean
SOD: Superoxide dismutase
SERM: Selective estrogen receptor modulator
TAMOX: Tamoxifen
TNF-α: Tumor necrosis factor alpha
WHO: World Health Organization
